# Obesity Accelerates Age-Associated Defects in Human B Cells Through a Metabolic Reprogramming Induced by the Fatty Acid Palmitate

**DOI:** 10.3389/fragi.2021.828697

**Published:** 2022-01-13

**Authors:** Daniela Frasca, Maria Romero, Denisse Garcia, Alain Diaz, Bonnie B. Blomberg

**Affiliations:** ^1^ Department of Microbiology and Immunology, University of Miami Miller School of Medicine, Miami, FL, United States; ^2^ Sylvester Comprehensive Cancer Center, University of Miami Miller School of Medicine, Miami, FL, United States

**Keywords:** obesity, aging, B cells, autoimmunity, metabolism

## Abstract

We have measured the secretion of autoimmune antibodies in plasma samples and in culture supernatants of blood-derived B cells from four groups of individuals: young lean (Y_L_), elderly lean (E_L_), young obese (Y_O_) and elderly obese (E_O_). We found secretion comparable in Y_O_ and E_L_ individuals, suggesting that obesity accelerates age-associated defects in circulating B cells. To define at least one possible molecular pathway involved, we used an *in vitro* model in which B cells from Y_L_ and E_L_ individuals have been stimulated with the Fatty Acid (FA) palmitate, the most common saturated FA in the human body. The rationale to use palmitate is that there is a chronic increase in circulating levels of palmitate, due to increased spontaneous lipolysis occurring during aging and obesity, and this may induce autoimmune B cells. Results herein show that *in vitro* incubation of B cells from Y_L_ and E_L_ individuals with the FA palmitate induces mRNA expression of T-bet, the transcription factor for autoimmune antibodies, as well as secretion of autoimmune IgG antibodies, with B cells from Y_L_ individuals looking similar to B cells from E_L_ individuals, confirming our initial hypothesis. The generation of autoimmune B cells in the presence of the FA palmitate was found to be associated with a metabolic reprogramming of B cells from both Y_L_ and E_L_ individuals. These results altogether show the critical role of the FA palmitate in inducing human B cell immunosenescence and show for the first time the importance of metabolic pathways in this process.

## Introduction

Aging is characterized by systemic chronic inflammation (inflammaging) (Franceschi et al., 2000), and is negatively associated with the production of protective antibodies to infectious agents and vaccines, and with increased production of autoimmune antibodies. Obesity, similar to aging, induces inflammaging which is responsible for functional impairment of immune cells and contributes to several chronic diseases typical of old age, such as cardiovascular disease (Apovian and Gokce, 2012), Type-2 Diabetes Mellitus (T2DM) (Hotamisligil, 2006; Shoelson et al., 2006; Johnson and Olefsky, 2013), cancer (Renehan et al., 2008), psoriasis (Setty et al., 2007), atherosclerosis (Casas et al., 2014), Alzheimer’s disease (Naderali et al., 2009; Singh-Manoux et al., 2018), and inflammatory bowel disease (Hass et al., 2006).

We have previously shown that secretion of autoimmune antibodies occurs in the human obese adipose tissue of adult individuals (Frasca et al., 2018; Frasca et al., 2020). These antibodies are specific for adipocyte-derived “self” proteins (AD) that are released following increased cell death due to hypoxia under obesity conditions (Frasca et al., 2018). We also found antibodies specific for malondialdehyde (MDA) (Frasca et al., 2021a), a measure of oxidative stress and lipid peroxidation occurring in the obese adipose tissue (Yesilbursa et al., 2005; Sankhla et al., 2012). Antibodies with these specificities are found increased in plasma samples of adults with obesity as compared to lean controls. We hypothesize that they will also be increased in the plasma of elderly individuals, and this may occur even in elderly lean individuals, due to the age-driven increased deposition of triglycerides on internal tissues and organs (liver, muscle, heart, pancreas, kidney) (Ryan and Nicklas, 1999; Machann et al., 2005; Saisho et al., 2007; Silaghi et al., 2008; Foster et al., 2011) as well as on blood vessels (Robert, 1999). The effect of aging on the secretion of autoimmune antibodies specific for AD and MDA, as well as the metabolic requirements of B cells secreting these antibodies, are still unknown.

In this study, we have measured the secretion of AD-specific and MDA-specific autoimmune antibodies in plasma samples and in culture supernatants of blood-derived B cells from four groups of individuals: young lean (Y_L_), elderly lean (E_L_), young obese (Y_O_) and elderly obese (E_O_) and characterized molecular pathways involved. The purpose of this study is to demonstrate that obesity accelerates age defects in human B cells. Because obesity is a condition associated with altered metabolism, we have characterized the metabolic phenotype of B cells. To define at least one possible mechanism, we have used an *in vitro* model in which B cells from Y_L_ and E_L_ individuals have been stimulated with the Fatty Acid (FA) palmitate. Palmitate is the salt of palmitic acid, the most common saturated FA in the human body, accounting for >60% of total saturated FAs in the body and >30% of total FAs in blood (Yu et al., 2012; Carta et al., 2017). The rationale is that there is a chronic increase in blood levels of the FA palmitate, due to increased spontaneous lipolysis occurring during aging and obesity, and this may induce autoimmune (pathogenic) B cells. Our results herein confirm this hypothesis and show that *in vitro* incubation of B cells from both Y_L_ and E_L_ individuals with the FA palmitate induces mRNA expression of T-bet, the transcription factor for autoimmune antibodies, and the secretion of AD-specific and MDA-specific IgG, similar to what is observed in cultures of B cells from E_L_ individuals. Moreover, the generation of autoimmune (pathogenic) B cells in the presence of the FA palmitate was found to be associated with a metabolic reprogramming of B cells from both Y_L_ and E_L_ individuals. These results altogether show the critical role of the FA palmitate in inducing human B cell immunosenescence and show for the first time the importance of metabolism in this process.

## Materials and Methods

### Participants

Participants were young (30–50 years) and elderly (≥65 years), both lean (BMI < 24.9) and obese (BMI ≥ 30), all recruited at the University of Miami Miller School of Medicine. All participants were healthy and were not using medications affecting the immune system. Subjects with type-2 diabetes mellitus (T2DM), autoimmune diseases, congestive heart failure, cardiovascular disease, chronic renal failure, malignancies, renal or hepatic diseases, infectious disease, trauma or surgery, pregnancy, or under substance and/or alcohol abuse were excluded from the study.

All participants signed an informed consent. The study was reviewed and approved by our Institutional Review Board (IRB, protocols #20070481 and #20160542), which reviews all human research conducted under the auspices of the University of Miami.

### PBMC Collection

Blood was drawn in Vacutainer CPT tubes (BD 362761). PBMC were isolated and cryopreserved. PBMC (1 × 10^6^/ml) were thawed and cultured in complete medium (c-RPMI, RPMI 1640, supplemented with 10% FCS, 10 μg/ml Pen-Strep, 1 mM Sodium Pyruvate, and 2 × 10^−5^ M 2-ME and 2 mM l-glutamine). After thawing the PBMC, viability was checked and samples were discarded if viability was <75%, as evaluated by trypan blue counting.

### B Cell Isolation and *In Vitro* Stimulation

B cells were isolated from thawed PBMC by magnetic sorting using CD19 Microbeads (Miltenyi) following manufacturer’s instructions. Cell preparations were typically >95% pure. For autoimmune antibody production, B cells were stimulated for 6 h-8 days in c-RPMI with CpG (InvivoGen ODN2006, 5 μg/10^6^ B cells in 1 ml) in the presence or absence of palmitate (final concentration 50 μM). Six or 24 h after stimulation, the mRNA was isolated and PCR reactions performed to measure expression of metabolic enzymes or the transcription factor T-bet, respectively. At day 8, supernatants were collected and IgG antibodies measured by ELISA.

### Naïve B Cell Sorting

PBMC were stained with Live/Dead detection kit (InVitrogen 1878898), anti-CD45 (Biolegend 368540), anti-CD19 (BD 555415), anti-CD27 (BD 555441) and anti-IgD (BD 555778) antibodies, and sorted using a FACS Aria (BD). Cell preparations were typically >98% pure. Naïve B cells (CD19+CD27-IgD+) were stimulated with CpG (5 μg/10^6^ B cells in 1 ml) in the presence of an AffiniPure F(ab′)_2_ fragment of goat anti-human IgG + IgM (anti-Ig) (2 μg/10^6^ B cells in 1 ml; Jackson ImmunoResearch Laboratories 109-006-127) for 1–10 days.

### Preparation of Palmitate

A stock solution of sodium palmitate (Sigma 408-35-5) was prepared at the concentration of 25 mg/ml in ethanol and kept at −80°C. On the day of the experiment, palmitate was thawed at 70°C, then resuspended in c-RPMI, vortexed and kept at 37°C in a water bath until the moment to be added to B cell cultures. Palmitate was used at the final concentration of 50 µM/10^6^ B cells in 1 ml. In a series of preliminary experiments we compared the effects of different doses of palmitate (50–200 µM/10^6^ B cells), but the doses of 100 and 200 µM induced significant cell death as evaluated by Trypan blue cell count. Therefore, all the experiments in this paper were performed using the physiologically relevant concentration of palmitate of 50 µM.

### ELISA to Measure Autoimmune Antibodies in Plasma and in Culture Supernatants

For AD-specific IgG antibodies, we isolated the adipocytes from the subcutaneous adipose tissue of patients undergoing weight reduction surgeries (bilateral breast reduction), as previously described (Frasca et al., 2018). After isolation, the adipocytes were centrifuged in a 5415C Eppendorf microfuge (2,000 rpm, 5 min). Total cell lysates were obtained using the M-PER (Mammalian Protein Extraction Reagent, ThermoFisher 78501), according to the manufacturer’s instructions. Aliquots of the protein extracts were stored at −80°C. Protein content was determined by Bradford (Bradford, 1976).

For MDA-specific IgG antibodies we used the MyBioSource MBS390120 kit.

Proteins and MDA were used at the concentration of 10 μg/ml in 1 × PBS to coat ELISA plates. After 1 h at room temperature, plates were washed, blocked with 1 × PBS containing 1% BSA (washing buffer) and then incubated for 30 min at 37°C. Then samples were added and incubated at room temperature for 3 h. Wells were washed thoroughly with washing buffer before receiving the detecting antibody goat anti-human IgG-Fc HRP-conjugated (Jackson ImmunoResearch 109-035-008, 1:5,000 diluted). After 1 h incubation at room temperature, wells were washed and given the substrate solution (TMB chromogen; Biosource SB01). Wells were incubated 15–20 min at room temperature to allow reactions to develop. Well contents were measured for absorbance at 405 nm.

### Flow Cytometry

To measure lipid uptake, PBMCs (2 × 10^6^/ml) were stained with the Deep Red Neutral Lipid Stain LipidTOX (Thermo Fisher H34476), for 30 min at room temperature, at the final concentrations recommended by the manufacturer. Cells were then washed and stained for 20 min at room temperature with anti-CD45, anti-CD19, as well as with the Live/Dead detection kit. Cells were washed and later acquired in a BD LSR Fortessa Flow cytometry instrument, using the APC channel to detect the signal from the LipidTOX. Fluorescence data were analyzed using FlowJo 10.5.3 software. In every experiment we included single color controls for compensation purposes as well as isotype control antibodies to set up the gates.

### mRNA Extraction and Quantitative (q)PCR

To evaluate RNA expression of the transcription factor T-bet, the mRNA was extracted from CpG-stimulated B cells, using µMACS magnetic beads (Miltenyi) following manufacturer’s instructions. The mRNA was eluted into 75 µl of pre-heated (65°C) elution buffer, and stored at −80°C until use. Reverse Transcriptase (RT) reactions were performed in a Mastercycler Eppendorf Thermocycler to obtain cDNA. Briefly, 10 µl of mRNA +10 µl of RT-mix were used for cDNA synthesis. Conditions were: 40 min at 42°C and 5 min at 65°C.

To evaluate RNA expression of enzymes involved in metabolic pathways, the mRNA was extracted from the same CpG-stimulated B cells, using the µMACS mRNA isolation kit, and reverse transcribed as indicated above.

Five µL of cDNA were used for qPCR. Reactions were conducted in MicroAmp 96-well plates and run in the ABI 7300 machine. Calculations were made with ABI software. For calculations, we determined the cycle number at which transcripts reached a significant threshold (Ct) for target genes and GAPDH (control). The difference in Ct values between GAPDH and the target gene was calculated as ΔCt. Then the relative amount of the target gene was expressed as 2^−ΔCt^ and indicated as qPCR values. All reagents were from Life Technologies. Taqman primers were: GAPDH, Hs99999905_m1; tbx21 (T-bet) Hs00894392_m1; HK2 (hexokinase-2), Hs00606086_m1; LDHA (lactate dehydrogenase), Hs01378790_g1; PDHX (pyruvate dehydrogenase), Hs00185790_m1; ACACB (Acethyl-CoA carboxylase), Hs01565914_m1.

### Mitostress Test

The metabolic profile of B cells from Y_L_ and E_L_ individuals, stimulated with CpG in the presence or absence of palmitate, was evaluated by the mitostress test and Seahorse technology that allows real-time evaluation of changes in oxygen consumption rates (OCR) and extracellular acidification rates (ECAR), measures of oxidative phosphorylation and of anaerobic glycolysis, respectively. The mitostress test was conducted in a Seahorse XFp extracellular flux analyzer (Agilent). Briefly, B cells at the concentration of 2.5 × 10^5^/well were seeded in a CellTAK (BD Biosciences)-coated plate for 20 min, and then incubated in XF DMEM medium supplemented with glutamine (2 mM), glucose (10 mM) and pyruvate (1 mM) for 45 min at 37°C. Maximal respiratory capacity was measured by treating with Oligomycin (1 μM) to block ATP production, followed by the uncoupling agent FCCP (fluoro-carbonyl cyanide phenylhydrazone, 5 μM), to dissipate proton gradients and allow electron transport and oxygen consumption to operate at maximal rate. This elevated OCR is suppressed by Rotenone/Antimycin (1 μM), showing that respiration is mitochondrial.

### Statistical Analyses

To examine differences between four groups, two-way ANOVA was used. Group-wise differences were analyzed afterwards with Bonferroni’s multiple comparisons test, with *p* < 0.05 set as criterion for significance. To examine relationships between variables, bivariate Pearson’s correlation analyses were performed. GraphPad Prism version 8.4.3 software was used to construct all graphs.

## Results and Discussion

### Effect of Aging and Obesity on the Secretion of Autoimmune IgG Antibodies

**FIGURE 1:**
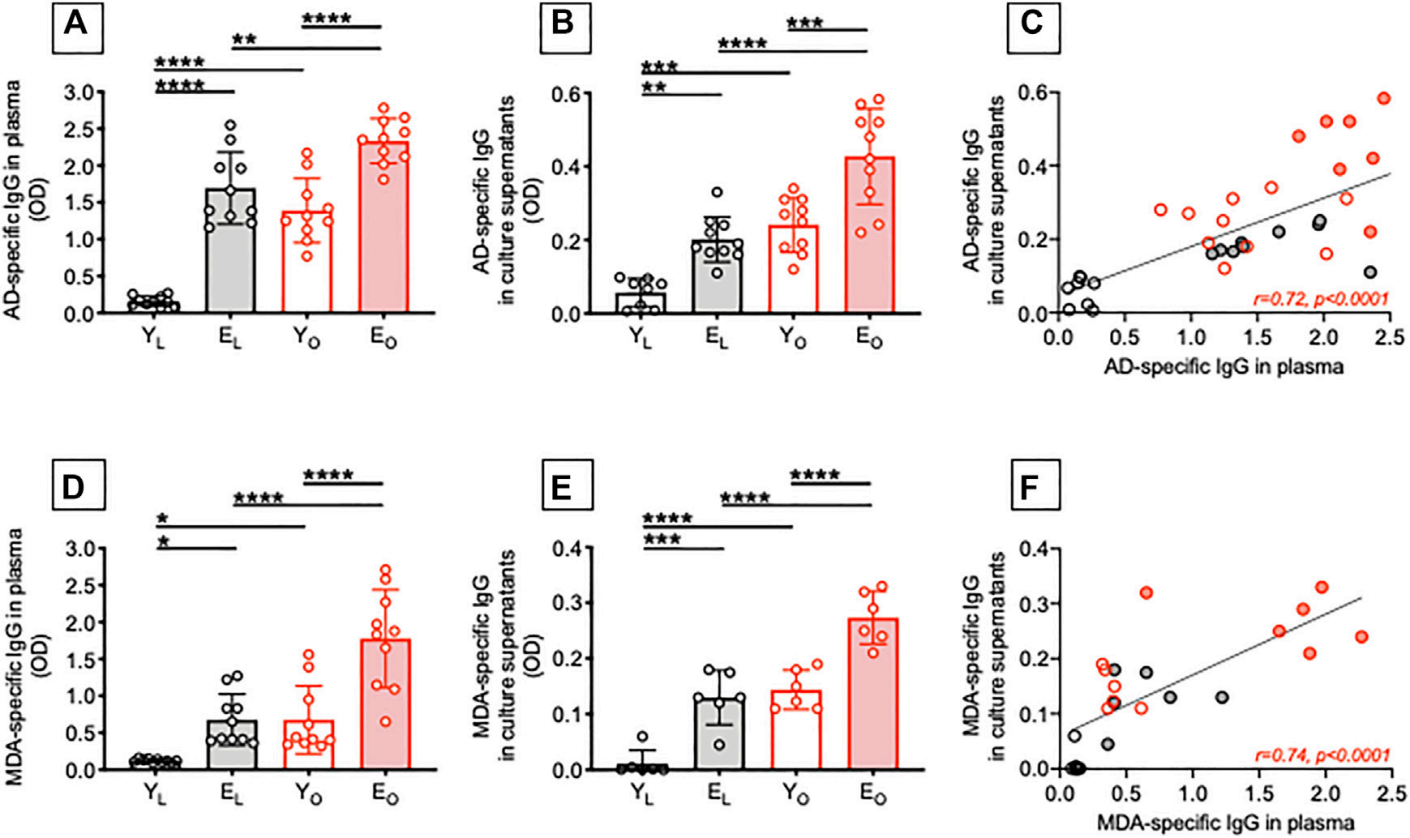
Effect of aging and obesity on the secretion of autoimmune IgG antibodies. Plasma samples were isolated from Y_L_, E_L_, Y_O_ and E_O_ individuals and analyzed by ELISA for the presence of AD-specific **(A)** or MDA-specific **(D)** IgG. B cells, isolated from the same individuals in A using magnetic beads, were stimulated for 8 days with CpG, supernatants were collected after 8 days and AD-specific **(B)** or MDA-specific **(E)** IgG were measured by ELISA. Correlations of AD-specific **(C)** and MDA-specific **(F)** IgG in plasma and culture supernatants. Mean comparisons between groups were performed by two-way ANOVA: *p < 0.05, **p < 0.01, ***p < 0.001, ****p < 0.0001. Correlations were calculated by bivariate Pearson’s correlation analyses.

We have previously shown that obesity, similar to aging, induces decreased B cell responses to the influenza vaccine in both young and elderly individuals (Frasca et al., 2016). We have also shown that secretion of IgG autoimmune antibodies occur in the obese adipose tissue of adult individuals (Frasca et al., 2018). Here, we compared the levels of IgG autoimmune antibodies specific for AD and MDA in plasma samples as well as in cultures of B cells isolated from the peripheral blood of Y_L_, E_L_, Y_O_ and E_O_ individuals. Results in Figure 1 show that plasma samples (A) and supernatants of B cell cultures (B) from E_L_ individuals are enriched in AD-specific IgG as compared to those from Y_L_ individuals. Moreover, in both young and elderly individuals, obesity induces a significant increase in autoimmune AD-specific IgG. The highest levels of these antibodies are in plasma and B cell cultures from E_O_ individuals. Importantly, the levels of AD-specific IgG are not different in E_L_ and Y_O_ individuals, suggesting that obesity per se induces age B cell defects that mirror those induced by aging, leading not only to reduced responses to the influenza vaccine but also to increased secretion of autoimmune antibodies. As expected, AD-specific IgG in plasma and in culture supernatants were positively associated (C).

Similar results were observed when we measured MDA-specific IgG antibodies. We found that aging, alone or together with obesity, induced a significant increase in autoimmune MDA-specific IgG antibodies in either plasma samples (D) and supernatants of B cell cultures (E), with these two measures being positively and significantly correlated (F).

To our knowledge, the presence of AD-specific and MDA-specific IgG antibodies has previously been reported by us in plasma (Frasca et al., 2021a) and in the adipose tissue of adult individuals with obesity (Frasca et al., 2018; Frasca et al., 2020). We are not aware of studies showing the presence of these autoimmune antibodies also in plasma/serum of elderly individuals, either lean or obese. Our results strongly support the concept that obesity accelerates B cell immunosenescence and drives the secretion of autoimmune antibodies.

### Effect of Aging and Obesity on Lipid Accumulation by B Cells

**FIGURE 2:**
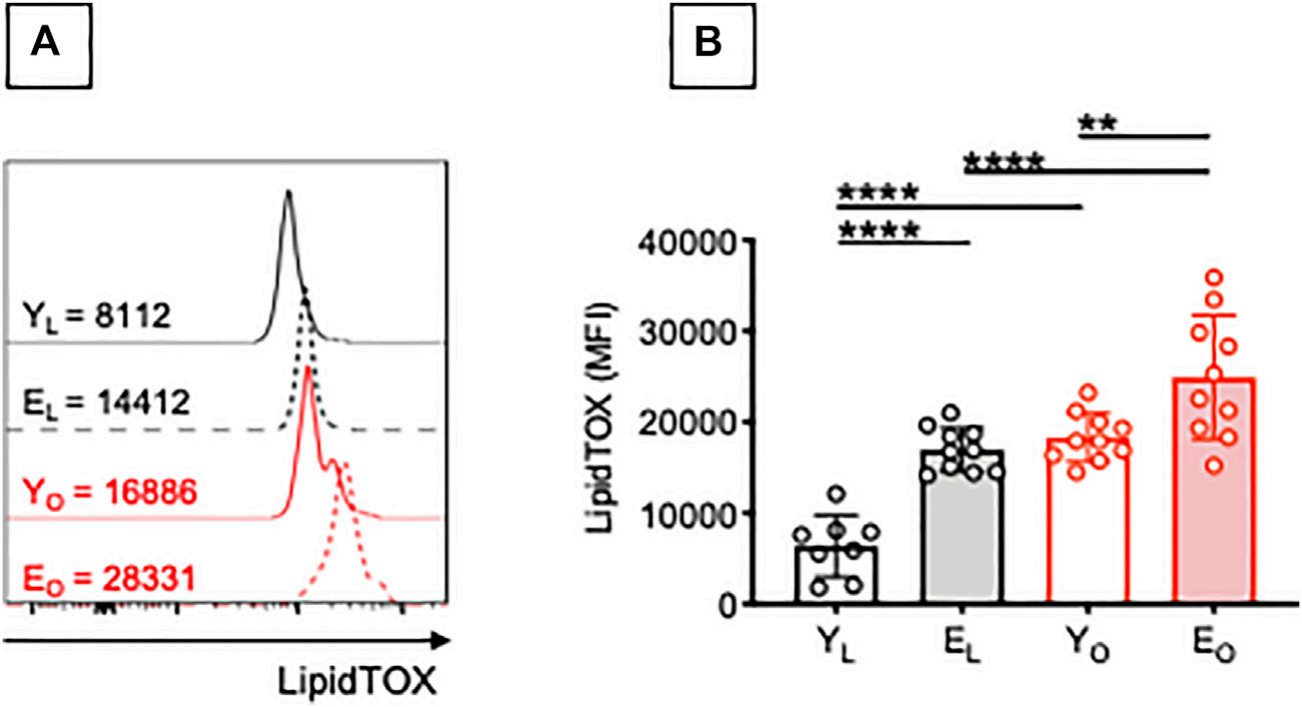
Effect of aging and obesity on lipid accumulation by B cells. PBMC were isolated from Y_L_, E_L_, Y_O_ and E_O_ individuals and stained first with the Deep Red Neutral Lipid Stain LipidTOX and then with anti-CD45, anti-CD19, and the Live/Dead detection kit. **(A)** MFI profiles of one representative donor/group. MFI values for negative controls (without the addition of LipidTOX are, respectively: 915 (Y_L_), 1214 (Y_O_), 837 (E_L_), 1025 (E_O_). **(B)** MFI data from all donors. Mean comparisons between groups were performed by two-way ANOVA: **p < 0.01, ****p < 0.0001.

Chronic elevation of circulating FAs is a hallmark of aging (Bonadonna et al., 1994; Tessari, 2000) and obesity (Jensen et al., 1989), and has been linked to the development of insulin resistance. In order to identify potential mechanisms involved in the secretion of AD-specific and MDA-specific IgG antibodies, we evaluated the capacity of B cells from Y_L_, E_L_, Y_O_ and E_O_ individuals to accumulate lipids. We have already shown that B cells from the blood and the adipose tissue of Y_O_ individuals spontaneously uptake lipids, as evaluated by staining with the neutral lipid stain LipidTOX (Frasca et al., 2021b). The rationale to look at lipids is because the obese adipose tissue is characterized by chronic lipolysis, the hydrolysis of tryglycerides to generate FAs and glycerol that are abundantly released in the circulation. Lipolysis is also occurring in the adipose tissue of E_L_ individuals (Robert, 1999; Ryan and Nicklas, 1999; Machann et al., 2005; Saisho et al., 2007; Silaghi et al., 2008; Foster et al., 2011), leading to the secretion of FAs in the circulation, as the tissue no longer stores them efficiently. Results in Figure 2 show that B cells from E_L_ individuals accumulate more lipids than those from Y_L_ individuals, as evaluated by staining with LipidTOX. Obesity induces a significant increase in lipid accumulation in B cells from both young and elderly individuals.

Little is known about the mechanisms involved in the accumulation of intracellular neutral lipids in B cells, and how this affects B cell function. It is known that lipid uptake by NK cells is associated with dysfunctional immunity against tumor cells (Michelet et al., 2018), whereas lipid uptake by dendritic cells completely inhibits their capacity to present antigens (Cubillos-Ruiz et al., 2015). Lipids may be utilized not only for metabolic but also for functional purposes. We hypothesize that lipid uptake by B cells triggers intrinsic inflammation and autoantibody secretion. FAs have indeed been shown to stimulate B cells to secrete inflammatory cytokines *in vitro* (Raval and Nikolajczyk, 2013).

### Palmitate *In Vitro* Stimulates the Secretion of AD-Specific Autoimmune Antibodies in B Cells From Both Y_L_ and E_L_ Individuals

**FIGURE 3:**
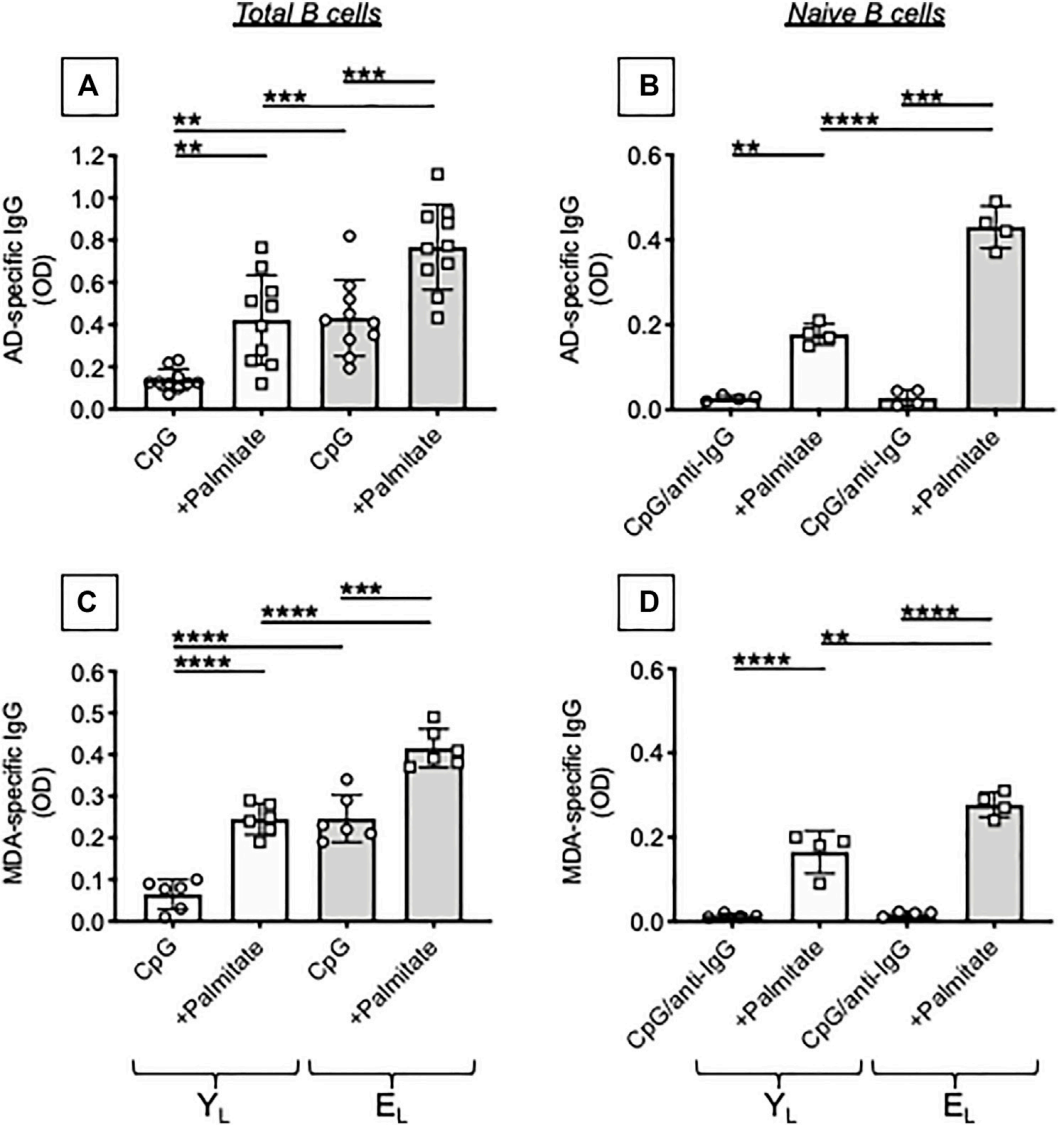
Palmitate *in vitro* stimulates the secretion of AD-specific autoimmune antibodies in B cells from both Y_L_ and E_L_ individuals. B cells, isolated from Y_L_ and E_L_ individuals using magnetic beads, were stimulated with CpG, alone or in the presence of palmitate, for 8 days. Then supernatants were collected and analyzed by ELISA for the presence of AD-specific **(A)** or MDA-specific **(C)** IgG. Naïve B cells, sorted from the blood of tha same Y_L_ and E_L_ individuals, were stimulated with CpG/anti-Ig, alone or in the presence of palmitate, for 8 days. Culture supernatants were tested by ELISA for the presence of AD-specific **(B)** or MDA-specific **(D)** IgG. Mean comparisons between groups were performed by two-way ANOVA: **p < 0.01, ***p < 0.001, ****p < 0.0001.

We tested the effects of adding the FA palmitate to cultures of CpG-stimulated B cells from Y_L_ and E_L_ individuals. Results in Figure 3 show that palmitate *in vitro* significantly increases the secretion of AD-specific antibodies (A) and MDA-specific antibodies (C) in culture supernatants of B cells from both Y_L_ and E_L_ individuals, with levels of IgG higher in E_L_ as compared to Y_L_. Interestingly, palmitate-induced secretion in cultures of B cells from Y_L_ individuals is similar to that observed in cultures of B cells from E_L_ individuals without addition of palmitate.

The composition of the B cell pool changes with age and increased frequencies of Double Negative (DN) B cells, the most pro-inflammatory B cell subset, have been reported by us (Frasca et al., 2016; Frasca et al., 2017a; Frasca et al., 2017b) as well as by other groups (Buffa et al., 2013; Martorana et al., 2014). We have previously shown that DN B cells secrete autoimmune antibodies (Frasca et al., 2019; Frasca et al., 2021a). In order to evaluate if naïve B cells were also contributing to the secretion of autoimmune antibodies in the presence of palmitate, we sorted naïve B cells and stimulated them *in vitro* with CpG + anti-Ig, in the presence or absence of palmitate. This stimulation of naïve B cells was used as a surrogate of antigen stimulation, because naïve B cells require the activation of BCR signal transduction to undergo class switch (Bernasconi et al., 2002). Results in Figure 3 show that, as expected, naïve B cells do not make AD-specific (B) or MDA-specific (D) autoimmune antibodies and this occurs in both Y_L_ and E_L_ individuals. However, the addition of palmitate induces the secretion of AD-specific IgG in naïve B cells from Y_L_ and even more in naïve B cells from E_L_ individuals, suggesting that palmitate makes naïve B cells able to class switch and secrete IgG with these autoimmune specificities.

### Palmitate *In Vitro* Increases mRNA Expression of T-Bet, the Transcription Factor for Autoimmune Antibody Production, in B Cells From Both Y_L_ and E_L_ Individuals

**FIGURE 4:**
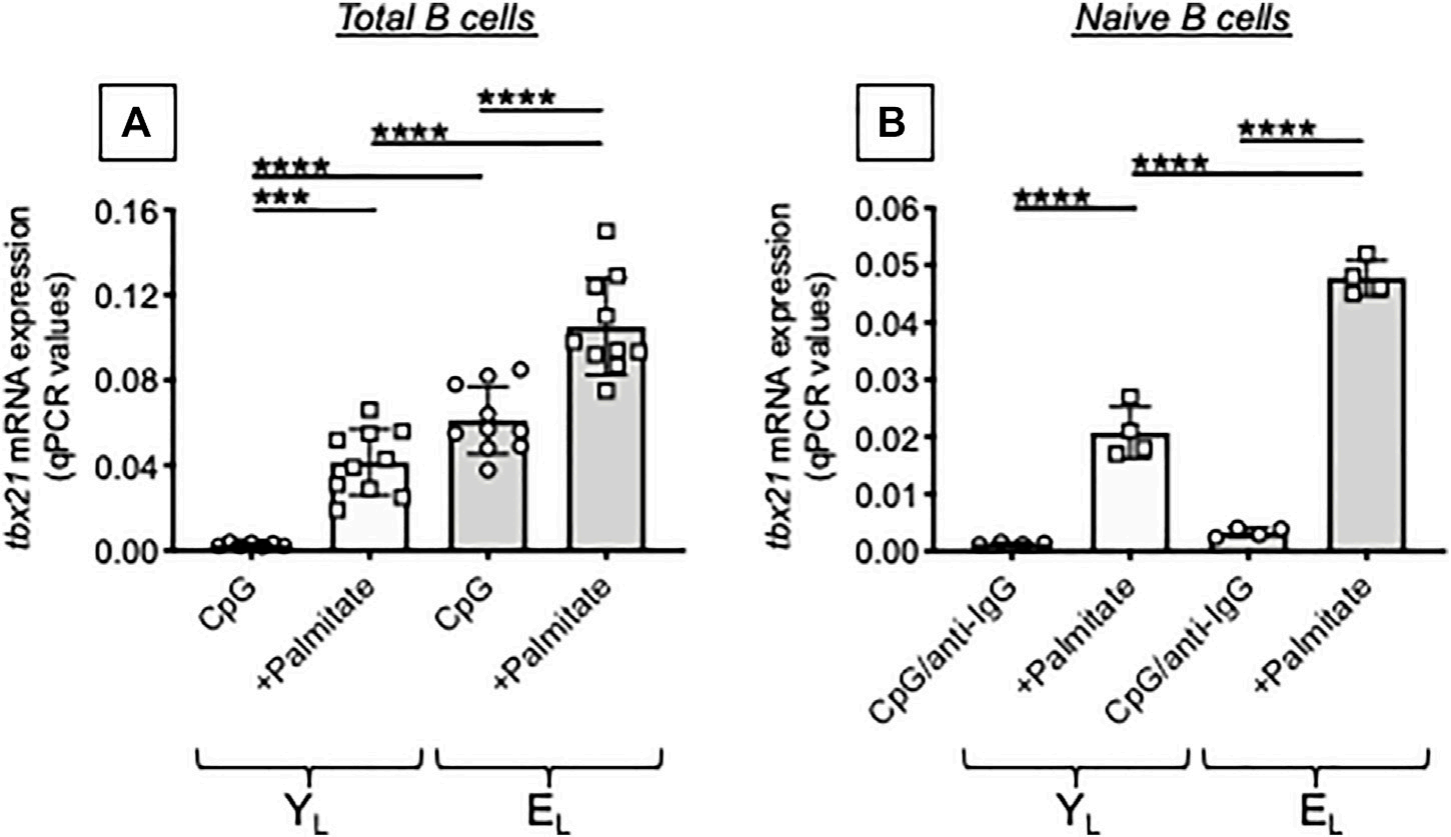
Palmitate *in vitro* increases mRNA expression of T-bet, the transcription factor for autoimmune antibody production, in B cells from both Y_L_ and E_L_ individuals. Total B cells **(A)** and naïve B cells **(B)**, isolated from the same Y_L_ and E_L_ individuals in Figure 3, were stimulated with CpG or CpG/anti-Ig respectively, alone or in the presence of palmitate, for 1 day. Then the mRNA was extracted and qPCR reactions run to evaluate the expression of tbx21. Results show qPCR values (2^−ΔCt^) of tbx21, normalized to GAPDH. Mean comparisons between groups were performed by two-way ANOVA: ***p < 0.001, ****p < 0.0001.

Next, we tested in the same B cell cultures mRNA expression of tbx21, the gene for the transcription factor of autoimmune antibody responses, T-bet (Myles et al., 2019). In agreement with the above results, the FA palmitate induced mRNA expression of tbx21, more in B cells from E_L_ as compared to B cells from Y_L_ individuals. Again, palmitate-induced tbx21 expression in cultures of B cells from Y_L_ individuals is similar to tbx21 expression in cultures of B cells from E_L_ individuals stimulated in the absence of palmitate (Figure 4A). When we tested naïve B cells, we found no mRNA expression of tbx21 in both Y_L_ and E_L_ individuals in the absence of palmitate which was able to induce tbx21 expression in naïve B cells from Y_L_ and even more in naïve B cells from E_L_ individuals (Figure 4B).

The results in Figures 3, 4 altogether demonstrate that palmitate accelerates age defects in B cells from Y_L_ individuals inducing the secretion of IgG antibodies with autoimmune specificities and mRNA expression of tbx21 similar to levels observed in E_L_ individuals. Our results have identified at least one mechanism through which obesity can drive secretion of autoimmune antibodies in aging. Briefly, FAs released in the adipose tissue reach the blood and stimulate T-bet activation in circulating B cells via pathways integrating signals from the BCR recognizing AD-derived antigens, from the TLRs recognizing FAs, and from receptors for cytokines increased by inflammaging, such as IFN-γ receptor.

Although it is already known that palmitate is involved in several pathological processes, including inflammatory and metabolic processes in different cell types, our results are the first to show an effect of palmitate on the induction of autoimmune B cells in both Y_L_ and E_L_ individuals. Direct exposure to palmitate is already known to induce the differentiation of CD4^+^ T cells into a detrimental, pro-inflammatory effector memory population, with these aberrant T cells being able to migrate to non-lymphoid inflammatory sites, and sustain low-grade chronic inflammation which is a hallmark of multiple metabolic disorders, including obesity and T2DM (Mauro et al., 2017). Palmitate also significantly increases signaling pathways associated with the secretion of pro-inflammatory cytokines in activated T cells (Zhou et al., 2019) as well as Th17 differentiation (Torres-Hernandez et al., 2020). A few studies also exist describing the pro-inflammatory effects of palmitate on macrophages (Kim et al., 2017; Karasawa et al., 2018; Korbecki and Bajdak-Rusinek, 2019), whereas exposure of NK cells to palmitate has been shown to induce loss of cytotoxic activity due to the loss of expression of both perforin and granzyme B (Michelet et al., 2018).

### Palmitate *In Vitro* Induces a Hyper-Metabolic Phenotype in B Cells From Both Y_L_ and E_L_ Individuals

The metabolic status of an individual strongly affects his/her immune system through nutrient supplies and cellular metabolism used by immune cells. This in turn regulates immune cell function, suggesting that an immunometabolic loop between systemic and cell intrinsic metabolism exists.

We have previously shown that B cells from both aged (Frasca et al., 2021c) and obese (Frasca et al., 2021b) individuals have a hyper-metabolic phenotype needed to satisfy the energetic demands associated with the secretion of pro-inflammatory cytokines and autoimmune antibodies. Therefore, we evaluated mRNA expression of several metabolic enzymes in cultures of CpG-stimulated B cells isolated from Y_L_ and E_L_ individuals, in the presence or absence of palmitate. We measured the following enzymes: HK2, hexokinase 2, a key glycolytic enzyme that phosphorylates glucose; LDHA, lactate dehydrogenase, that converts pyruvate into lactate and represents a measure of anaerobic glycolysis; PDHX, a component of the pyruvate dehydrogenase complex that converts pyruvate into acetyl-CoA and represents a measure of oxidative phosphorylation and mitochondrial function; ACACB, Acethyl-CoA carboxylase, a regulator of FA synthesis.

**FIGURE 5:**
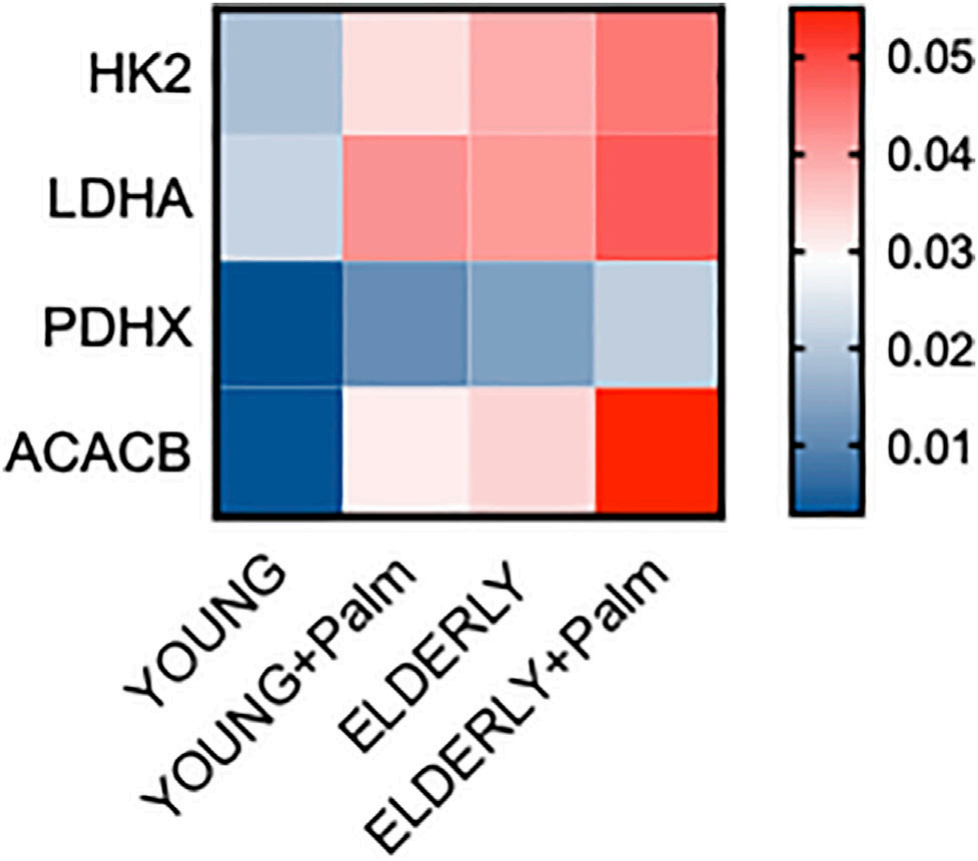
Palmitate *in vitro* induces a hyper-metabolic phenotype in B cells from both Y_L_ and E_L_ individuals. Total B cells from the same Y_L_ and E_L_ individuals in Figures 3, 4 were stimulated with CpG, alone or in the presence of palmitate for 6 h, and evaluated for the mRNA expression of HK-2, LDHA, PDHX and ACACB. Heatmap shows qPCR values (2^−ΔCt^) of metabolic markers, normalized to GAPDH, from four individuals/group.

**FIGURE 6:**
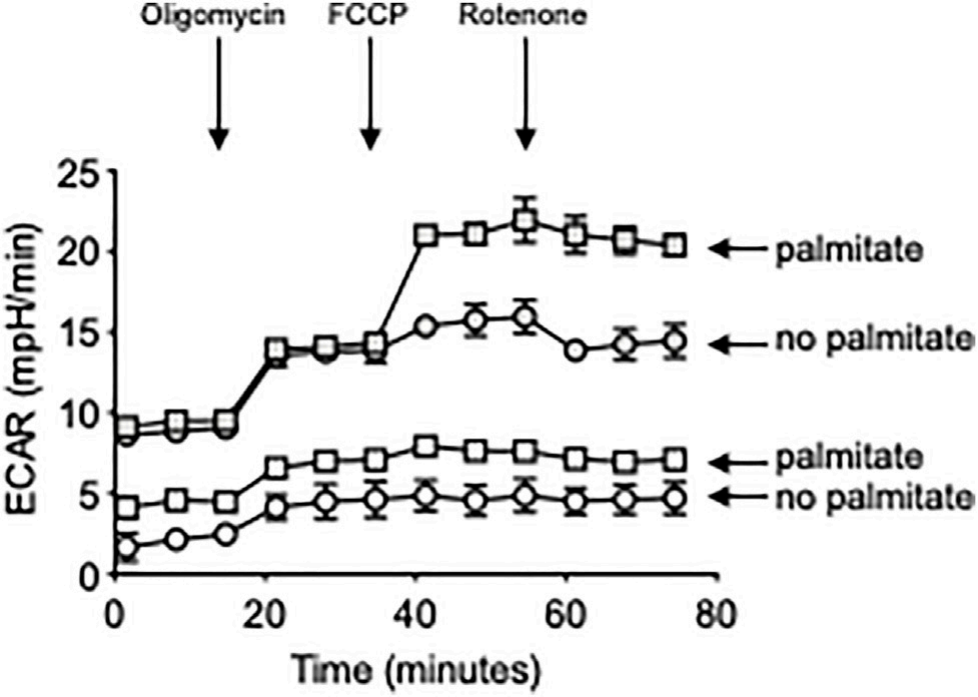
Palmitate *in vitro* induces higher ECAR in B cells from both Y_L_ and E_L_ individuals. Total B cells from the same Y_L_ and E_L_ individuals in Figures 3, 4 were stimulated with CpG, alone or in the presence of palmitate for 6 h, and seeded into the wells of an extracellular flux analyzer at the concentration of 2 × 10^5^/well in triplicate and run in a mitostress test. Bottom two groups refer to: B cells from Y_L_ individuals. Top two groups refer to: B cells from E_L_ individuals.

Results in Figure 5 show a palmitate-induced increase in the RNA expression of all the metabolic enzymes but especially of ACACB, involved in the metabolism of FAs, and LDHA. Consistent with a metabolic switch to glycolysis, we also found a significant increase in ECAR in CpG-stimulated B cells from both Y_L_ and E_L_ individuals, *in vitro* treated with palmitate (Figure 6), whereas OCR were not modified (not shown). The lack of effect of palmitate on OCR, also confirmed by the minimal increase in the expression of PDHX, may be dependent on the palmitate-induced mitochondrial respiratory dysfunction, similar to what has already been described for macrophages (Chen et al., 2019) and myoblasts (Patková et al., 2014). These results altogether suggest that palmitate induces a shift to anaerobic glycolysis in B cells from Y_L_ and E_L_ individuals, an essential pathway for the expansion of pathogenic B cells, and provide previously uninvestigated metabolic mechanisms for the secretion of autoimmune IgG antibodies. These results also suggest a novel means of regulating pathogenic function of B cells.

## Conclusion and Future Directions

Results herein show that the chronic increase in blood levels of the FA palmitate that spontaneously occur during aging/obesity may be responsible for the induction of pathogenic B cells that secrete autoimmune antibodies. Indeed, we showed that *in vitro* incubation of B cells from Y_L_ individuals with the FA palmitate induces mRNA expression of T-bet, the transcription factor for autoimmune antibodies, and the secretion of AD-specific and MDA-specific IgG, similar to what is observed in cultures of B cells from E_L_ individuals. We also showed that B cells stimulated in the presence of the FA palmitate mainly engage in metabolic pathways such as anaerobic glycolysis, an essential pathway necessary for the expansion of pathogenic B cells.

Our results present for the first time not only the effects of the FA palmitate on B cells, but also FA metabolism as a mechanistic insight into B cell function and autoimmune antibody secretion. The potential role of the FA palmitate as a metabolic immune modulator underscores the importance of further studies evaluating the effects of lipids not only on B cells but also on other immune cell types. Once these effects will be known, it will be possible to develop new therapeutic strategies acting directly on metabolic pathways to promote a better immune system.

## Data Availability

The original contributions presented in the study are included in the article/Supplementary Material, further inquiries can be directed to the corresponding author.
